# Preoperative predictive factors affecting sentinel lymph node positivity in breast cancer and comparison of their effectiveness with existing nomograms

**DOI:** 10.1097/MD.0000000000032170

**Published:** 2022-12-02

**Authors:** Cengiz Ceylan, Hikmet Pehlevan Ozel, Ibrahim Agackiran, Buket Altun Ozdemir, Hakan Atas, Ebru Menekse

**Affiliations:** a Department of Surgery, Inönü University, Malatya, Turkey; b Department of Surgery, Ankara City Hospital, Ankara, Turkey; c Department of Surgery, Elaziğ Fethi Sekin City Hospital, Elazıital, Elaziğ, Turkey.

**Keywords:** breast cancer, nomogram, risk estimation, sentinel lymph node dissection

## Abstract

This study aimed to establish a strong regression model by revealing the preoperative predictive factors for sentinel lymph node (SLN) positivity in patients with early stage breast cancer (ESBC). In total, 445 patients who underwent SLN dissection for ESBC were included. All data that may be potential predictors of SLN positivity were retrospectively analyzed. Tumor size >2 cm, human epidermal growth factor receptor 2 (HER2) + status, lymphovascular invasion (LVI), palpable tumor, microcalcifications, multifocality or multicentricity, and axillary ultrasonographic findings were defined as independent predictors of SLN involvement. The area under the receiver operating characteristic (ROC) curve (AUC) values were 0.797, 0.808, and 0.870 for the Memorial Sloan-Kettering Cancer Center (MSKCC) nomogram, MD Anderson Cancer Center (MDACC) nomogram, and our regression model, respectively (*P* < .001). The recent model for predicting SLN status in ESBC was found to be stronger than existing nomograms. Parameters not included in current nomograms, such as palpable tumors, microcalcifications, and axillary ultrasonographic findings, are likely to make this model more meaningful.

## 1. Introduction

Axillary nodal status is an important prognostic factor of early stage breast cancer (ESBC).^[1]^ Sentinel lymph node biopsy (SLNB) is the standard safe surgical approach for axillary staging in patients with ESBC with clinically negative lymph nodes.^[2]^ Although SLNB reduces the risk of morbidity associated with axillary lymph node dissection (ALND), it cannot be completely eliminated. After SLNB, complications such as lymphedema, and sensory deficits occur at a rate of 6 to 11% postoperatively.^[3]^ Currently, completion of ALND is safely omitted in the presence of 1 or 2 positive sentinel lymph node (SLN) in treated patients, since the reliability of 5 year disease-free survival rates has been demonstrated according to the results of the American College of Surgeons Oncology Group (ACOSOG) Z0011 trial.^[3]^ Also, an ongoing randomized controlled multicenter Dutch trial was conducted to investigate whether SLNB can be safely omitted in clinically node negative-breast cancer (BC) patients treated with breast conserving therapy.^[4]^ However, axillary staging still seems to remain important for planning adjuvant therapy and deciding whether to receive radiotherapy in patients with ESBC treated with mastectomy. Therefore, models to preoperatively predict axillary involvement in clinically node negative-BC that will decrease morbidity and cost by reducing invasive axillary intervention have gained importance. Two computed nomograms where performed using the *Breast Cancer Nomogram to Predict Positive Sentinel Lymph Nodes, without Neoadjuvant Chemotherapy* generated by MD Anderson Cancer Center (MDACC) nomogram, and the other was a *breast cancer nomogram: sentinel lymph node metastasis* generated by Memorial Sloan-Kettering Cancer Center (MSKCC) nomogram to predict the probability of a positive SLN. These nomograms use various clinicopathological variables to predict the SLN positivity.^[5,6]^ Previous studies have attempted to increase the predictive power of these nomograms for SLN positivity by adding various clinical data to existing models or supporting them with imaging methods.^[7,8]^

The aim of this study was to establish a strong regression model by revealing the predictive factors of SLN positivity in ESBC patients in a wider perspective and compare the strength of the model with the nomograms generated by the MSKCC, MDACC to estimate the preoperative SLN.

## 2. Methods

### 2.1. Patient population

This study was approved by the institutional review board (approval number: 2363) of our institution and conducted in accordance with the declaration of Helsinki. Patients who did not have clinical axillary lymph node involvement were operated on for ESBC and underwent SLNB between January 1st, 2010 and December 31st, 2018 were included in our study. Demographic and clinicopathological data were obtained from the Ankara City Hospital system.

In clinical staging, patients with locally advanced and metastatic BC, those receiving neoadjuvant chemotherapy, those with a history of BC or other malignancies, and those who had undergone axillary or breast-related interventions that would cause reactive lymph node hyperplasia before breast imaging were excluded from the study.

### 2.2. SLN technique and determination of groups

After the patient was induced with anesthesia and prepared for surgery in a sterile manner, 10 mL of patent blue was injected subareolar. After injection of patent blue the breast was massaged for 10 min. The skin and subcutaneous tissue was passed with a 2 cm incision from 2 cm inferior to the end of the axillary hairs. Following excision, blue-stained lymph nodes were sent to be frozen. Frozen sections were evaluated under a microscope by hematoxylin and eosin (H & E) staining. Axillary dissection was decided for those with positive results. Micrometastases detected in the excised lymph nodes were considered negative. Patients were divided into 2 groups: those with and without SLN involvement, regardless of the number of lymph nodes involved.

### 2.3. Clinical, pathological, and radiological evaluation

For BC diagnosis and treatment planning, all patients underwent with bilateral breast ultrasonography (USG) before surgery and patients aged ≥40 years were evaluated using bilateral mammography. In the evaluation of the axillary region by USG, lymph nodes with normal or slightly thick hilum (<3 mm), without loss of hilum were considered as cN0 and SLN biopsy was performed. Lymph nodes with a thick cortex but also round appearance and loss of hilum on USG were not included in the study because they were considered pathological.

The patients’ pathological data included age, physical examination status of the tumor, tumor size, tumor histology, estrogen receptor (ER) status, progesterone receptor (PR) status, human epidermal growth factor receptor 2 (HER2) status, histological grade status, presence of in situ lesions accompanying invasive carcinoma, lymphovascular invasion (LVI) and Ki-67 score, breast density, presence of microcalcifications on mammography, Breast Imaging Reporting and Data System (BI-RADS), multifocality or multicentricity, quadrant of tumor location, and ultrasonographic characteristics of the axillary lymph nodes. Immunohistochemical staining for ER ≥1% and PR ≥1% was considered positive.

Breast density was classified in accordance with the Breast Imaging Reporting and Data System (BI-RADS). According to this system, breast density was defined as follows: category A = almost entirely fat, category B = scattered fibroglandular densities, category C = heterogeneously dense, and category D = extremely dense.^[9]^

Ki-67 score was defined as the percent of Ki-67 positive cells measured in 1000 cancer cells by immunohistochemistry. The score of Ki-67 was provided continuous (in the range of 0–100 corresponding to the percentage of positive tumors cells) and categorical (first cutoff point 7.5% and second cutoff point 14%).

The relationship between SLN involvement and the data obtained was analyzed. Moreover, the estimated percentage of SLN involvement in patients was determined using nomograms generated by the MSKCC and MDACC, which are used to predict SLN involvement in patients with BC.

### 2.4. Statistical analysis

The minimum number of patients required to complete our study, which was computed using the odds ratios obtained through the logistic regression analysis, with 95% confidence level and 90% power, was a total of 362 patients (181 for each group).

Normality of distribution was tested using the Kolmogorov–Smirnov test. Equivalent nonparametric tests were used (Mann–Whitney *U* test). The mean, standard deviation, median, minimum and maximum values of the variables are presented. The ideal cutoff point for tumor size and Ki-67 score was determined by receiver operating characteristic (ROC) curve analysis. A chi-square analysis was conducted for categorical variables. The frequency and percentage values of these variables are presented. Prospective selective multivariate logistic regression analysis was performed with the variables found to be statistically significant. The Hosmer–Lemeshow test was used to evaluate the goodness of fit. To assess the success of the logistic regression model, ROC curve analysis was conducted and performance measures were computed. In the definition of the predictive model, the area under the ROC curve (AUC) values were specified as follows; 0.90 to 1.00 = Excellent, 0.80 to 0.90 = Good, 0.70 to 0.80 = Fair, 0.60 to 0.70 = Poor, 0.50 to 0.60 = Failed. Statistical significance was set at *P* < .05.

## 3. Results

A total of 445 female patients were included in this study. SLN involvement was observed in 194 patients (43.60%). The mean age of all patients was determined to be 53.71 ± 11.93 (21–85). A mean of 3.86 ± 1.62 (1–13) SLN was excised from all patients. A mean of 3.96 ± 1.70 (1–13) lymph nodes was dissected from 251 patients with negative SLN, while a mean of 3.73 ± 1.49 (2–10) lymph nodes was dissected from 194 patients with metastatic SLN.

The difference between the continuous values of tumor size and Ki-67 score percentage between patients with and without SLN involvement was statistically significant (*P* < .001, *P* < .01) (Table 1).

**Table 1:** General characteristics of patients and tumors by sentinel lymph node tumor (continuous numerical data).

	Sentinel lymph node involvement (n, %)	Mean ± SD	*P*
Age	Absent (251, 56.4%)	54.64 ± 11.99	.089
	Present (194, 43.6%)	52.50 ± 11.78	
Tumor size (cm)	Absent (251, 56.4%)	1.77 ± 1.00	**<.001** *
	Present (194, 43.6%)	2.41 ± 0.99	
ER (%)	Absent (251, 56.4%)	52.78 ± 37.79	.370
	Present (194, 43.6%)	50.31 ± 36.79	
PR (%)	Absent (251, 56.4%)	50.21 ± 38.11	.372
	Present (194, 43.6%)	46.37 ± 37.71	
Ki-67 score (%)	Absent (116, 55.2%)	15.59 ± 20.06	**.005** *
	Present (94, 44.8%)	19.98 ± 18.94	

ER = estrogen receptor, PR = progesterone receptor.

* P < .05.

Cutoff values were calculated for continuous data such as tumor size and Ki-67 expression, affecting SLN involvement. Tumor size >2, Ki-67 score ≥7.5 and ≥14 cutoff values were found to be the ideal cutoff points for SLN involvement (Table 2).

**Table 2:** The success of nomograms on our sample, with ideal cutoff performance for tumor size and Ki-67 score.

	Cutoff value	Sensitivity	Selectivity	AUC	95% CI
Tumor size (cm)	>2	58.80%	71.70%	0.695	0.646–0.744
Ki-67 score (%)	≥7.5	73.40%	51.70%	0.612	0.535–0.689
	≥14	51.10%	63.80%		

AUC = area under the receiver operating characteristic (ROC) curve, CI = confidence interval.

The difference between the categorical data of tumor size, tumor histology, HER 2 receptor, LVI, histological grade, physical examination, microcalcification, multifocality or multicentricity, axillary ultrasonographic findings, Ki-67 score ≥7.5, and Ki-67 score ≥14 between patients with and without SLN involvement was statistically significant (*P* < .001 for all except Ki-67 score ≥7.5 and *P* = .031 for Ki-67 score ≥14) (Tables 3 and 4).

**Table 3:** General characteristics of patients by sentinel lymph node tumor (categorical data).

Features of patients		Sentinel lymph node involvement		***P***
		Absent (n, %)	Present (n, %)	
Age	<65	199, 55.4%	160, 44.6%	.398
	≥65	52, 60.5%	34, 39.5%	
Breast density*	A	42, 53.2%	37, 46.8%	.617
	B	99, 60.4%	65, 39.6%	
	C	88, 55.0%	72, 45.0%	
	D	22, 52.4%	20, 47.6%	
Quadrant location of the tumor	Central	8, 66.7%	4, 33.3%	.195
	Upper inner	40, 70.2%	17, 29.8%	
	Upper outer	154, 54.2%	130, 45.8%	
	Lower inner	17, 50.0%	17, 50%	
	Lower outer	32, 55.2%	26, 44.8%	
Physical examination	Non-palpable	91, 79.8%	23, 20.2%	**<.001** *
	Palpable	160, 48.3%	171, 51.7%	
Microcalcification	Absent	222, 81.9%	49, 18.1%	**<.001** *
	Present	29, 16.7%	145, 83.3%	
Multifocality/multicentrity	Absent	210, 66.7%	105, 33.3%	**<.001** *
	Present	41, 31.5%	89, 68.5%	
Axillary ultrasonographic findings	Normal finding	214, 62.9%	126, 37.1%	**<.001** *
	Thick cortex	37, 35.2%	68, 64.8%	

* Breast density; category A = almost entirely fat; category B = scattered fibroglandular densities; category C = heterogeneously dense; and category D = extremely dense.

* P < .05.

**Table 4:** General characteristics of tumors by sentinel lymph node tumor (categorical data).

Features of tumors		Sentinel lymph node involvement		*P*
		Absent (n, %)	Present (n, %)	
Tumor size	≤2 cm	180, 69.2%	80, 30.8%	**<.001** *
	>2 cm	71, 38.4%	114, 61.6%	
Tumor histology	IDC	188, 54.3%	158, 45.7%	**<.001** *
	ILC	5, 27.8%	13, 72.2%	
	Special types*	36, 83.7%	7, 16.3%	
	Other†	22, 57.9%	16, 42.1%	
ER (%)	Negative	53, 57.6%	39, 42.4%	.794
	Positive	198, 56.1%	155, 43.9%	
PR (%)	Negative	51, 56.7%	39, 43.3%	.955
	Positive	200, 56.3%	155, 43.7%	
Ki-67 score (≥7.5%)	Negative	60, 70.6%	25, 29.4%	**<.001** *
	Positive	56, 44.8%	69, 55.2%	
Ki-67 score (≥14%)	Negative	74, 61.7%	46, 38.3%	**.031** *
	Positive	42, 46.7%	48, 53.3%	
HER2	Negative	219, 61.2%	139, 38.8%	**<.001** *
	Positive	32, 36.8%	55, 63.2%	
Triple negativity	No	222, 55.4%	179, 44.6%	.180
	Yes	29, 65.9%	15, 34.1%	
Accompanying DCIS or LCIS	Absent	96, 60.8%	62, 39.2%	.169
	Present	155, 54.0%	132, 46.0%	
LVI	Absent	220, 75.6%	71, 24.4%	**<.001** *
	Present	31, 20.1%	123, 79.9%	
Histological grade	I	88, 73.3%	32, 26.7%	**<.001** *
	II	109, 50.7%	106, 49.3%	
	III	54, 49.1%	56, 50.9%	

DCIS = Ductal carcinoma in situ, ER = estrogen receptor, HER2 = human epidermal growth factor receptor 2, IDC = invasive ductal carcinoma, ILC = invasive lobular carcinoma, LVI = lymphovascular invasion, LCIS = lobular carcinoma in situ, PR = progesterone receptor.

* Special types: papillary carcinoma, mucinous carcinoma, tubular carcinoma;

† Other types: medullary carcinoma, cribriform carcinoma, adenoid cystic carcinoma, micropapillary carcinoma, apocrine carcinoma, and microinvasive carcinoma ≥1% were considered positive.

* P < .05.

Univariate logistic regression analyses were conducted, considering SLN involvement as the dependent variable. Following the prospective selective multivariate logistic regression analysis performed with the variables that were found to be statistically significant as a result of these analyses, the variables of tumor size, HER2 receptor, LVI, physical examination, microcalcification, multifocality or multicentricity and axillary ultrasonographic findings were found to be statistically significant. Based on the results of the Hosmer–Lemeshow test, it was determined that the obtained logistic regression model had a good fit with the data (C = 5.315, *P* = .723).

According to the established model, the risk of SLN involvement increases 2443 times in tumors >2 cm in size (*P* = .006). Likewise, it was found that this risk increased 3169 times in HER2 positive individuals (*P* = .005) compared to those with negativity, while the risk increased 12,441 times in patients with LVI compared to those without (*P* < .001) and it increased 2276 times in palpable tumors compared to non-palpable tumors (*P* = .043). Furthermore, the presence of microcalcifications on mammography increased the risk of SLN involvement by 26,559 times (*P* < .001). This risk increased by 2351 times in multifocal or multicentric tumors (*P* = .016). Lastly, the presence of a thick cortex in the lymph node on axillary USG increases the risk of SLN involvement 2367 times (*P* = .024) (Table 5).

**Table 5:** Multivariate analysis of clinical and pathological characteristics associated with sentinel lymph node involvement.

Characteristics	Odds ratio	95% CI	*P*
Tumor size >2 cm	2.443	1.297–4.603	**.006** *
HER2 positivity	3.169	1.421–7.067	**.005** *
Presence of LVI	12.441	6.246–24.779	**<.01** *
Palpable tumor	2.276	1.028–5.037	**.043** *
Presence of microcalcification	26.559	13.444–52.469	**<.01** *
Presence of multicenter tumor	2.351	1.173–4.715	**.016** *
Thick cortex lymph node	2.367	1.121–5.000	**.024** *

CI = confidence interval, HER2 = human epidermal growth factor receptor 2, LVI = lymphovascular invasion.

* P < .05.

**Figure 1.**
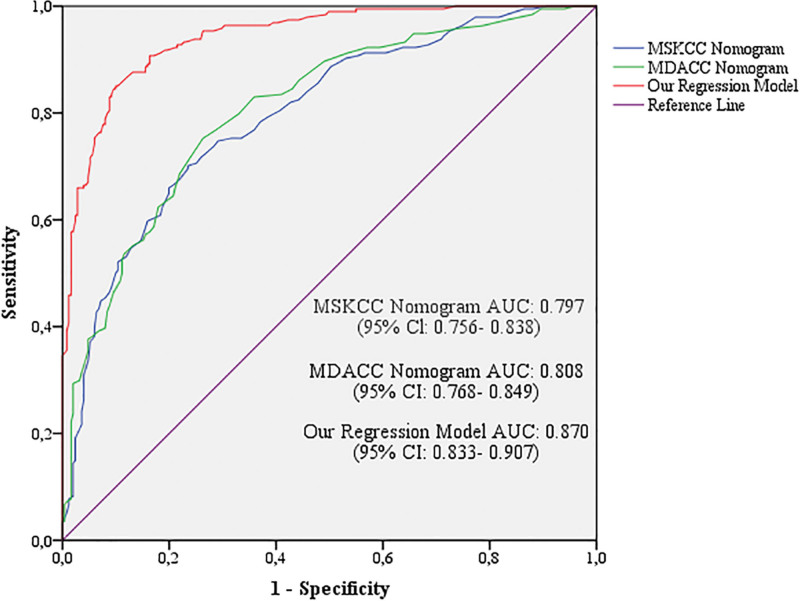
ROC curves of current nomograms and our regression model. ROC = receiver operating characteristic.

The sensitivity of the established multivariate logistic regression model in determining SLN involvement was 84.00%, its selectivity was 90.00%, the correct classification rate was 87.42%, the positive predictive value was 86.70%, and the negative predictive value was 87.94%. The area under the ROC curve for the recent study was 0.870 (95% CI: 0.833–0.907), while those for MSKCC and MDACC were 0.797 (95% CI: 0.756–0.838) and 0.808 (95% CI: 0.768–0.849). This value was higher than the MSKCC and MDACC nomogram scores (*P* < .001) (Fig. 1).

## 4. Discussion

SLNB provides axillary staging with high accuracy and low morbidity, thus preventing unnecessary ALND and related morbidities in most patients.^[3]^ Due to morbidities, surgery was not performed in the axilla. Therefore, studies in which the axilla was followed by USG were planned for patients with tumor size ≤2 cm and no pathological lymph nodes in the axilla.^[10]^ Also positron emission tomography (PET)/magnetic resonance imaging (MRI) will lead to the role of PET/MRI in axillary staging of BC patients.^[11]^ These studies also pioneered the development of nomograms for SLN prediction. In contrast 2 nomograms developed for the prediction of SLN involvement in BC used in our study are MDACC and MSKCC nomograms.^[5,6]^ In a study by Veerapong et al, young age, tumor histology, tumor size, multifocality, and presence of LVI were considered as positive predictive factors. Upper inner quadrant and triple-negative hormone status were negative predictive factors for axillary involvement.^[5]^ In a study by Bevilacqua et al, a correlation was found between age, tumor size, tumor histology, LVI, tumor localization, multifocality, ER and PR status, and SLN metastasis. In this study, localization of the tumor in the upper inner quadrant was shown to be a negative predictive factor. Although the presence of ER and PR was low, it increased SLN metastasis.^[6]^

The risk factors for SLN metastasis in patients with BC and the results of the validation study of the MSKCC nomogram by Qiu et al were as follows: SLN positivity was observed in nearly 33% of 1227 patients. In this study, the AUC value of the MSKCC nomogram score was 0.730.^[12]^

In our study, the areas under the ROC curve of the risk percentages of the MSKCC and MDACC nomograms were 0.797 and 0.808, respectively. The area under the ROC curve of the established regression model was determined to be 0.870, which was higher than that of most previously generated nomograms. Furthermore, tumor size of >2 cm, HER2 receptor status, LVI, tumor palpability on physical examination, presence of microcalcifications on mammography, multicentric or multifocal tumor characteristics and axillary lymph node characteristics on ultrasound were identified as independent risk factors for sentinel lymph involvement. In our study, unlike other nomograms, the presence of palpable tumors, microcalcifications, and lymph node features on USG were found to be independent parameters.

Some studies have stated that palpable tumors manifest varying clinical and pathological features compared to non-palpable tumors.^[13]^ It has been suggested in the literature that palpability of the tumor on physical examination, regardless of tumor size, is an independent predictor of axillary nodal involvement.^[13]^ In a study by Chao et al on ESBC, it was demonstrated that there was a significant difference in lymph node metastasis between palpable tumors and non-palpable tumors (*P* < .001).^[14]^ In our study, palpable tumors increased the probability of lymph node metastasis in regression analysis (*P* = .043).

The presence of microcalcifications on mammography has been shown to be a poor prognostic marker for BC.^[14]^ Also in studies investigating the relationship between breast USG, mammography, and BC molecular subtypes, Rashmi et al found that HER2 overexpression was higher in the presence of microcalcification.^[15]^ Similarly, in a study by Cen et al, a statistical correlation was found between microcalcification status and HER2 and luminal A subtype.^[16]^ In our study, microcalcification increased the risk of SLN involvement (*P* < .001) and was the strongest independent predictive factor affecting SLN involvement. Microcalcifications are considered to be a poor prognostic feature in BC and as a result of our study, we also present microcalcifications into the literature as a new parameter affecting SLN involvement.

In the study by Qui et al, in which the demographic and histopathological data used in nomograms, as well as the characteristics of axillary ultrasound and axillary lymph nodes, were used, the transverse diameter of the lymph node (*P* = .044), cortical thickness (*P* = .002), hilum loss (*P* = .001), clinical tumor size (*P* = .018), histological grade of the tumor (*P* < .001) and ER (*P* = .001) in multivariate analyses were found to be independent predictive factors for axillary lymph node metastasis and were used in the model. In the analyses of the performance of the established model, the AUC was determined to be 0.864.^[17]^

In a study seeking an answer to the theory about the increase in lymph node metastasis rate in ER and PR positivity cases or the low rate of lymph node metastasis in triple-negative tumors, it was revealed that histological grade, mitotic score and nuclear pleomorphism increased as a result of increased hormone receptor positivity.^[18]^ It was shown in a study that patients with negative HER2 and ER status had lower lymph node metastasis rates.^[19]^ In our study, no significant relationship was found between lymph node metastasis in the ER (*P* = .794) and PR (*P* = .955) groups.

In a study by Mendez et al on BC, 95.1% of patients had invasive ductal carcinoma and 4.9% of them had specific subtypes. SLN positivity rates were 4.1% for special subtypes and 34.2% for invasive ductal carcinoma.^[20]^ In our study, lymph node metastasis was not detected in any of the 12 patients with tubular carcinoma, which is one of the special types. There was a significant inverse correlation between special subtypes and lymph node metastasis compared to patients with invasive ductal carcinoma (*P* < .001).

A significant difference was observed in terms of SLN involvement according to the Ki-67 score (*P* = .005). In addition, groups were generated based on cutoff values (≥7.5 and ≥14) previously defined in the literature and the Ki-67 score was found to be significant in terms of SLN involvement (*P* < .001 and *P* < .031). Although the cutoff value of Ki-67 was determined to be ≥14 at the St. Gallen meeting, Koyama et al determined the cutoff value of Ki-67 as ≥7.5 in their study and found that the statistical difference between the groups generated based on this value was more significant in terms of SLN involvement (*P* = .019).^[21]^

In our study, an inclusive regression model including physical examination characteristics, radiological characteristics, and pathological characteristics of the patients was established to predict axillary nodal involvement in BC patients without early stage clinical axillary lymph node involvement, and it was demonstrated that the established model is better than previously developed prediction models for the same purpose. This is probably related to the addition of new breast- and lymph node-related parameters that affect SLN involvement in the parameters of existing nomograms. This prediction model could be helpful for physicians in the clinically accurate prediction of SLN metastasis, prognosis of patients, and in management of treatment process.

## Acknowledgments

The authors wish to thank all the staff of the surgery, radiology and pathology departments in Ankara City Hospital.

## Author contributions

**Conceptualization:** Cengiz Ceylan, Ebru Menekse.

**Data curation:** Ibrahim Agackiran, Hakan Atas, Buket Altun Ozdemir.

**Investigation:** Hakan Atas.

**Methodology:** Buket Altun Ozdemir.

**Project administration:** Cengiz Ceylan.

**Supervision:** Ebru Menekse, Hikmet Pehlevan Ozel.

**Writing – original draft:** Cengiz Ceylan.

**Writing – review & editing:** Cengiz Ceylan, Ebru Menekse.
